# Time course of acid–base regulation at high‐altitude: A century of insight

**DOI:** 10.1113/EP093365

**Published:** 2025-11-10

**Authors:** Andrew R. Steele, Jordan D. Bird, Michael M. Tymko

**Affiliations:** ^1^ Centre for Heart, Lung and Vascular Health, School of Health and Exercise Sciences University of British Columbia–Okanagan Kelowna British Columbia Canada; ^2^ Division of Critical Care Medicine, Department of Medicine, Vancouver General Hospital University of British Columbia Vancouver British Columbia Canada; ^3^ Integrative Cerebrovascular and Environmental Physiology SB Laboratory, Department of Human Health Sciences, College of Biological Science University of Guelph Guelph Ontario Canada

## 1 INTRODUCTION

Initial exposure to hypoxia – whether hypobaric (e.g., high‐altitude; >2500 m) or normobaric (e.g., $F_{iO_{2}}$ < 15%) – results in a reduction in arterial oxygen content ($C_{aO_{2}}$). The first defence against this reduction in $C_{aO_{2}}$ is the hypoxic ventilatory response, which progressively intensifies over time at altitude (Severinghaus et al., 1963). After a week or so, an increase in haemoglobin concentration also acts to improve – and often normalize – $C_{aO_{2}}$. However, the sustained hyperventilation lowers arterial carbon dioxide ($P_{\mathrm{aC}O_{2}}$) and persists even after haemoconcentration has restored $C_{aO_{2}}$ beyond low altitude levels (Barcroft et al., 1923; Severinghaus et al., 1963). The mismatch between metabolic CO_2_ production and the elevated ventilatory drive raises pH (i.e., respiratory alkalosis), prompting the kidneys to excrete bicarbonate [HCO_3_^−^] to help re‐establish acid–base balance (Severinghaus et al., 1963).

In this context, the recent study by Skalla et al. (2026) examined acid–base balance and fluid regulation during the early stages of high‐altitude acclimatization. Twelve unacclimatized participants (5 females; mean age = 50 years) were rapidly transported from 575 to 3100 m. Arterial blood gases ($P_{\mathrm{aC}O_{2}}$, $P_{aO_{2}}$, pH and [HCO_3_^−^]_a_), electrolytes (chloride, calcium, potassium, sodium), renin, urine output and urine composition (osmolarity, sodium, potassium, chloride) were assessed after 24 and 44 h at high‐altitude. The authors reported that, as might be expected, hyperventilation at 24 h induced respiratory alkalosis, with only modest reductions in [HCO_3_^−^]_a_ and hence metabolic compensation. By 44 h, renal [HCO_3_^−^]_a_ excretion increased, partially correcting the respiratory alkalosis, with arterial pH trending towards low altitude values but still remaining elevated. Urine output also increased during early high‐altitude acclimatization, rising from 1.4 mL kg^−1^ h^−1^ in the first 24 h to 2.3 mL kg^−1^ h^−1^ in the subsequent 20 h. The authors attributed this increase in urinary output to hypoxic diuresis. Here, we aim to extend the discussion of Skalla et al. (2026) by situating their findings within the broader findings of high‐altitude acid–base research, highlighting unresolved questions, and offering an alternative interpretation of the potential hypoxic diuresis observed.

## 2 CURRENT GAPS IN ACID–BASE CHARACTERIZATION DURING EARLY HIGH‐ALTITUDE ACCLIMATIZATION

**FIGURE 1:**
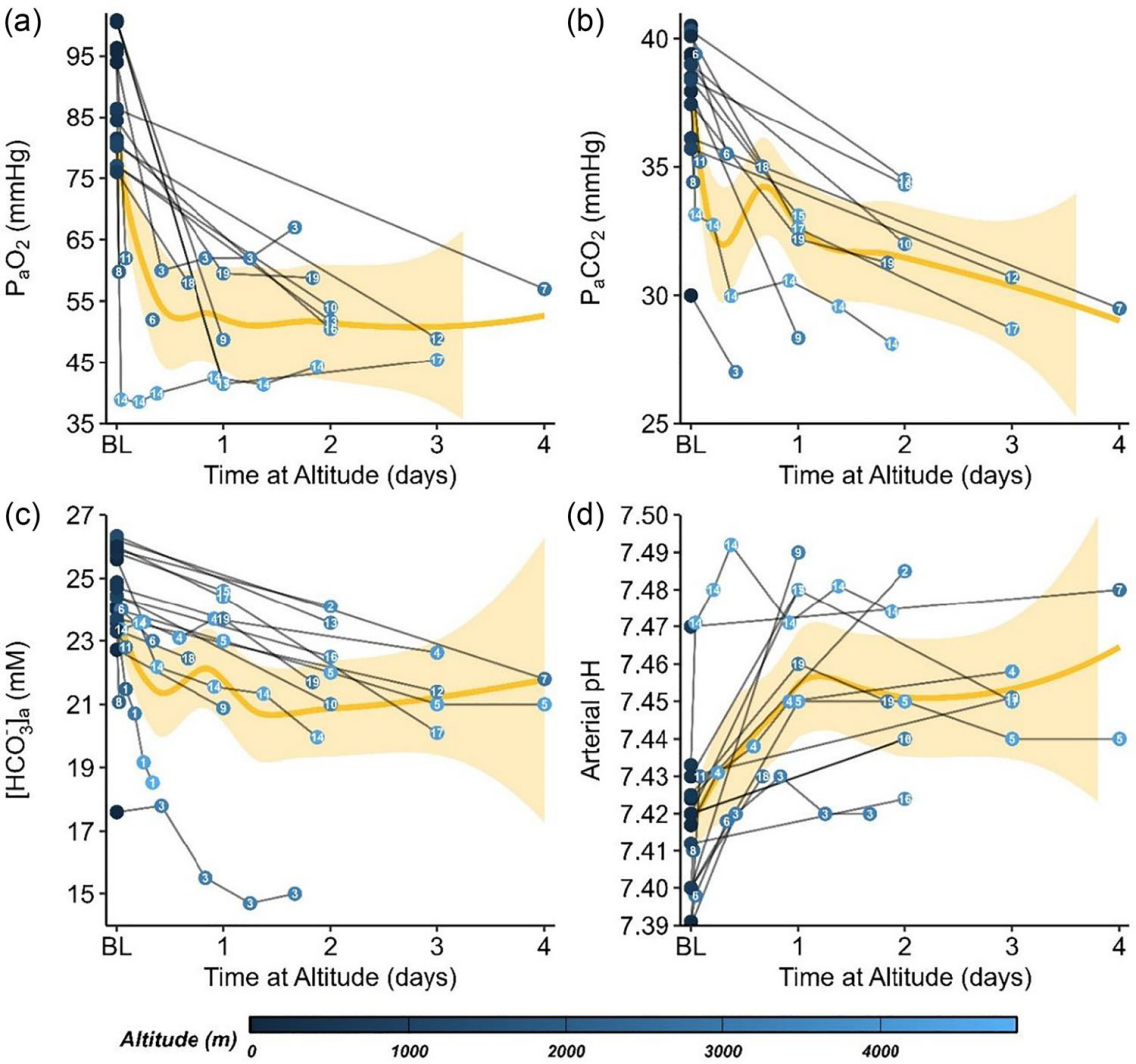
Compilation of studies on arterial acid–base regulation within the first 4 days of hypobaric hypoxia exposure. Data are compiled from *N* ≈ 338 participants across 19 studies. Locally estimated scatterplot smoothing from weighted averages by study *n* with 95% confidence intervals were fit to the data (gold line). Studies are presented as name, sample size, and altitude. Studies: (1) Hurtado et al. (1948), *n* ≈ 12, 2390/3140/4540/4860 m; (2) Severinghaus et al. (1963), *n* = 6, 3800 m; (3) Vogel & Harris (1967), *n *= 16, 3353 m; (4) Moncloa et al. (1970), *n* = 12, 4300 m; (5) Lenfant et al. (1971), *n* = 4, 4509 m; (6) Dempsey et al. (1974), *n* = 7, 3100 m; (7) Frayser et al. (1975), *n* = 8, 2926 m; (8) Dillard et al. (1995), *n* = 9, 2438 m; (9) Imray et al. (2003), *n* = 10, 3459 m; (10) Burgess et al. (2004), *n* = 14, 3446 m; (11) Nakano et al. (2015), *n* = 7, 3000 m; (12) Zouboules et al. (2018), *n* = 18, 3440 m; (13) Hoiland et al. (2019), n = 21, 3400 m; (14) Biollaz et al. (2021), *n* = 18, 4559 m, arterialized earlobe; (15) Steele et al. (2020), *n* = 24, 4330 m; (16) Bird et al. (2021), *n* = 16, 3800 m; (17) Steele et al. (2022), *n* ≈ 15, 4300 m; (18) Furian et al. (2022), *n* = 109, 3100 m; (19) Skalla et al. (2026), *n* = 12, 3100 m.

A century ago, Barcroft et al. (1923) conducted a seminal study in the Peruvian Andes, reporting that arterial pH increased following weeks of high‐altitude exposure. More recently, a meta‐analysis synthesized evidence from 41 studies (>550 participants) that measured arterial pH during the first 3 days of hypobaric hypoxia exposure, many conducted around 3000–4000 m, highlighted in Figure 1 (Forrer et al., 2023). These data indicate that [HCO_3_^−^]_a_ excretion stabilizes within the first 48 h of high‐altitude acclimatization and remains largely unchanged thereafter. For example, even after 10 months at 3233 m, respiratory alkalosis persisted (Porcelli et al., 2017). Based on previously published work, it is clear that arterial pH and [HCO_3_^−^]_a_ become stable somewhere between 24 and 48 h. To better determine the exact time point at which acid–base status stabilizes at high‐altitude, a future study could consider serial blood sampling at shorter (hourly) intervals to capture the incomplete normalization of pH and the kinetics of [HCO_3_^−^] excretion. However, obtaining multiple arterial blood samples over such a short time frame could be logistically challenging and would likely require placement of an arterial catheter to avoid repeated punctures. Nevertheless, progress in this field depends on building upon the foundational studies that have carefully defined the physiological time course of high‐altitude acclimatization in earlier work.

## 3 FLUID BALANCE DURING EARLY HIGH‐ALTITUDE ACCLIMATIZATION

We acknowledge the difficulties of controlling fluid balance in field studies and the authors’ efforts to record water intake to calculate fluid balance. However, their conclusion that ‘*during early acclimatization diuresis outweighs fluid shifts in the generation of altitude‐associated plasma volume contraction*’ is not well supported. In contrast, Roche et al. (2022), at a simulated altitude of 3500 m, found that with standardized fluid and sodium intake, urinary flow briefly increased between 0 and 6 h but was unchanged from 6 to 24 h, while 24‐h urinary output and total body water were unchanged at 24 h and remained stable for 4 days despite a decrease in plasma volume. These findings suggest that early haemoconcentration is driven by fluid redistribution between the plasma and interstitial space, rather than hypoxic diuresis. Both Roche et al. (2022) and Skalla et al. (2026) observed decreased circulating renin concentrations, suggesting a possible mechanism for hypoxic diuresis. However, it would seem that the greater urinary output in Skalla et al. (2026) is more likely explained by increased fluid and/or sodium intake rather than a direct hypoxic effect per se. Overall, fluid redistribution remains the most plausible driver of early plasma volume contraction, and attributing this response to hypoxic diuresis is not widely supported.

## 4 CONCLUSION

Although the novelty of the findings is unclear (as highlighted in Figure 1), we commend the authors for conducting a study that involves considerable logistical challenges and adding a complementary data set to the existing available literature on acute acid–base balance and fluid homeostasis during early acclimatization to high‐altitude. Future studies should consider: (i) better characterizing the 24–48 h window of acclimatization, when [HCO_3_^−^] is likely to change most substantially, partially reversing respiratory alkalosis; (ii) applying a comprehensive acid–base framework such as the Stewart approach, which incorporates $P_{\mathrm{aC}O_{2}}$, weak acids (e.g., albumin) and strong ion differences (e.g., Na⁺, K⁺, Cl^−^), which may better represent pH at high‐altitude due to physiological changes (e.g., decreases in circulating proteins); and (iii) standardizing salt and fluid intake in the field, particularly during chronic hypoxia (e.g., ∼1 month).

## AUTHOR CONTRIBUTIONS

Andrew R. Steele, Jordan D. Bird, and Michael M Tymko. all contributed to the conception and drafting of the manuscript and approved the final version. All authors have read and approved the final version of this manuscript and agree to be accountable for all aspects of the work in ensuring that questions related to the accuracy or integrity of any part of the work are appropriately investigated and resolved. All persons designated as authors qualify for authorship, and all those who qualify for authorship are listed.

## CONFLICT OF INTEREST

None declared.

## FUNDING INFORMATION

Jordan D. Bird is funded through a Canadian Institute of Health Research – Doctoral Award (no. 187576). Michael M. Tymko is funded by a NSERC discovery grant (40165).
